# MicroRNA Associated with the Invasive Phenotype in Clear Cell Renal Cell Carcinoma: Let-7c-5p Inhibits Proliferation, Migration, and Invasion by Targeting Insulin-like Growth Factor 1 Receptor

**DOI:** 10.3390/biomedicines10102425

**Published:** 2022-09-28

**Authors:** Thomas J. Kalantzakos, Luke E. Sebel, James Trussler, Travis B. Sullivan, Eric J. Burks, Carmen D. Sarita-Reyes, David Canes, Alireza Moinzadeh, Kimberly M. Rieger-Christ

**Affiliations:** 1 Department of Translational Research, Lahey Hospital & Medical Center, Burlington, MA 01805, USA; 2 Department of Urology, Lahey Hospital & Medical Center, Burlington, MA 01805, USA; 3 Department of Pathology & Laboratory Medicine, Boston University School of Medicine, Boston Medical Center, Boston, MA 02118, USA

**Keywords:** clear cell renal cell cancer (ccRCC), microRNA, insulin like growth factor 1 receptor (IGF1R), proliferation, migration, invasion

## Abstract

Differential microRNA (miRNA) expression can portend clear cell renal cell carcinoma (ccRCC) progression. In a previous study, we identified a subset of dysregulated miRNA in small renal masses, pT1 ccRCC (≤5 cm) that are associated with an aggressive phenotype. The present study investigated miRNA expression in clinical stage I (cT1) tumors (≤5 cm), comparing pathologic stage I (pT1) tumors to those upstaged to pathologic stage 3 (pT3) after surgery following identification of renal vein invasion or invasion into adjacent fat tissue within Gerota’s fascia. Twenty cT1 tumors were examined in an miRNA screening, 10 pT1 and 10 pT3 tumors. The ccRCC cell lines 786-O and Caki-1 were used to assess the impact of let-7c-5p and its protein target insulin-like growth factor 1 receptor (IGF1R). Cells were transfected with pre-let-7c-5p and assessed through cell proliferation, migration, and invasion assays. IGF1R expression was evaluated through Simple Western, and interaction between let-7c-5p and IGF1R was confirmed via luciferase reporter assay. Screening identified 20 miRNA, including let-7c-5p, that were dysregulated between pT1 and pT3 upstaged tumors. This miRNA was also downregulated in our previous study of pT1 tumors that progressed to metastatic disease. Transfection of ccRCC cells with pre-let-7c-5p significantly inhibited proliferation, migration, invasion, and IGF1R expression. These findings suggest that miRNA dysregulation is involved in ccRCC progression, specifically through invasion, and that let-7c-5p downregulation contributes to the aggressiveness of small ccRCC tumors, in part, through its regulation of IGF1R.

## 1. Introduction

Renal cell carcinoma (RCC) is a heterogeneous group of cancer variants arising from a variety of specialized cells along the length of the nephron [1]. Clear cell renal cell carcinoma (ccRCC) is the most common histologic subtype of RCC, accounting for 75–80% of cases [1]. ccRCC is disproportionately represented within metastatic RCC, comprising over 90% of cases [2]. One area of particular interest centers on small renal masses, which have seen a disproportionate rise in incidence through the rise of cross-sectional imaging [3]. Clinical staging of incidentally discovered ccRCC tumors relies on radiographic size criteria and gross invasion of venous structures or within Gerota’s fascia [4]. However, microscopic renal vein and perirenal fat invasion is not adequately detected through imaging, prompting an upstaging of clinical stage I (cT1) tumors to pathologic T3a (pT3a) at the time of surgery [5]. This phenomenon has been identified in about 5% of patients and is associated with a substantial increase in mortality risk [5].

Recent studies have concentrated on the identification of novel prognostic indicators to improve treatment algorithms [5]. The potential of microRNA (miRNA) as an identifiable biomarker in ccRCC tumors has previously been demonstrated in many instances, including our previous study characterizing miRNA in pathologic stage I (pT1) tumors that progressed to metastatic disease [6]. miRNAs are small, highly conserved non-coding RNA molecules that bind to the three prime untranslated regions (3′UTR) of mRNA transcripts, silencing gene expression [7]. miRNAs exert their influence through targeting important signaling pathways and regulatory networks [8]. In addition to its prognostic value, miRNA dysregulation has been identified as a driver of metastasis and disease progression in ccRCC [9], and has been associated with early relapse after nephrectomy in RCC patients [10]. We previously investigated small pT1 ccRCC tumors (pT1 ≤ 5 cm) that ultimately progressed to metastatic disease [6]. One particular miRNA which corresponded to poorer cancer-specific survival was let-7c-5p, which was downregulated in aggressive small renal masses.

The prognostic value of let-7c-5p expression in ccRCC and the conferral of aggressiveness through its downregulation in other cancers has been established. In paired ccRCC tissue specimens, let-7c was downregulated in ccRCC tissue compared to adjacent normal [11]. Expression of let-7c was also demonstrated to be significantly reduced in aggressive early metastatic ccRCC tumors compared with non-metastatic [9]. Additionally, elevated levels of let-7c-5p in the urine can be used to differentiate RCC patients from healthy controls [12]. Let-7c-5p has also been established as a tumor suppressor in a wide range of other cancers [13,14,15,16,17,18]. In non-small cell lung cancer, for example, a significant association was demonstrated between low let-7c-5p expression and metastasis, venous invasion, advanced TNM stages, and poor survival [17].

A miRNA exerts its influence on cellular phenotype through the specific protein targets that it interacts with. These interactions exist as part of a complex network, where each miRNA can silence hundreds of unique targets, while each protein is regulated by many miRNAs [19]. The role of a particular miRNA in this network and in tumorigenesis can be elucidated by first confirming the individual targets with which they interact. Algorithmic methods, based on predicted base pairing stability, can be used to identify potential protein targets [20], while experimental methods can be used to verify those targets [19]. This was the approach we used to investigate the role of let-7c-5p in clinical stage I ccRCC.

One protein target that is predicted to interact with let-7c-5p is the insulin-like growth factor 1 receptor (IGF1R) [21,22,23,24,25]. IGF1R is a transmembrane receptor with tyrosine kinase activity when bound to insulin-like growth factor 1 (IGF-1) that shares high structural homology with the insulin receptor [26]. A study in head and neck squamous cell carcinoma has experimentally validated the interaction between IGF1R and let-7c-5p, connecting silenced expression of IGF1R to decreased proliferation, migration, and epithelial-mesenchymal transition (EMT) [27]. The expression levels of let-7c-5p have also shown a correlation with IGF1R through Western blotting in dental pulp-derived mesenchymal stem cells [28] and stem cells from the apical papilla [29]. IGF1R knockdown has been demonstrated to produce a similar impact in ccRCC cells. Aberrant IGF1R expression has been demonstrated to contribute to the malignant transformation of renal cells through its impact on cell proliferation, dedifferentiation, and apoptosis [26]. Additionally, siRNA-induced knockdown of IGF1R has been demonstrated to abrogate cell migration and invasion [30,31].

IGF1R expression levels within ccRCC have been shown to be prognostic with respect to patient outcomes. Patients with high IGF1R expression experience significantly poorer cancer-specific survival, with a 70% increased risk of death [32]. IGF1R staining correlates with Fuhrman grading in ccRCC tissues [33]. Furthermore, IGF1R overexpression corresponds to poor prognosis in other cancers, such as breast cancer, where it regulates EMT [34].

The objective of this study is to identify alterations in miRNA expression profiles of clinical T1 ccRCC that are characteristic of pathologic upstaging to pT3 at the time of surgery. In addition, we seek to better understand the role of let-7c-5p in ccRCC. Although this miRNA has been identified as a biomarker of aggressiveness in ccRCC and associated with the invasive phenotype in other tumor types, its specific mechanisms of action in ccRCC are not clearly delineated. Therefore, we aimed to further understand the role of let-7c-5p expression on the proliferation, migration, and invasion of ccRCC cells, along with its potential interaction with IGF1R. We investigated the link between let-7c-5p and IGF1R in order to improve our understanding of how each factor influences the progression of small ccRCC tumors and contributes to local invasion.

## 2. Materials and Methods

### 2.1. Samples

This study was performed according to the guidelines and regulations of the Lahey Hospital & Medical Center (LHMC) Institutional Review Board (IRB) protocol 2002-080 and the Boston Medical Center (BMC) IRB protocol H-37859. Although localized tumors as large as 7 cm can be classified as stage I, this study was limited to tumors ≤5 cm with the goal of characterizing the miRNA expression of small renal masses with or without an invasive phenotype. Twenty-four aged and ISUP grade-matched patients with cT1 renal masses who had undergone partial or radical nephrectomy for ccRCC at Lahey Hospital and Medical Center and Boston Medical Center were identified. Ten pT1 cases and ten pT3 cases were included in this study. Two patients were excluded due to the preoperative presence of metastasis, while two others were excluded due to synchronous tumors. A pathologist specializing in urologic malignancies (EJB & CSR) reviewed all samples to confirm clear cell histology, pathologic stage I or III disease, and assign an ISUP grade.

### 2.2. RNA Isolation and miRNA Screening Analysis of Clinical Samples

RNA was isolated from patient samples using the Qiagen FFPE AllPrep RNA isolation kit (Qiagen, Hilden, Germany) and was analyzed for quantity and purity (OD 260/280 ratio) using the Epoch spectrophotometer (BioTek, Winooski, VT, USA). Screening analysis was performed using Human miRNome panels I&II version 5 (Qiagen), analyzing 752 miRNAs via qRT-PCR according to the manufacturer’s protocol.

### 2.3. Cell Culture

The human ccRCC cell lines 786-O (ATCC^®^ CRL-1932™) and Caki-1 (ATCC^®^ HTB-46™) (American Type Culture Collection, Manassas, VA, USA) were cultured under standard conditions (37 °C, 5% CO_2_). A VHL mutant primary epithelial clear cell adenocarcinoma with altered HIF and VEGF pathways [35], 786-O, was grown in RPMI 1640 (ATCC). Caki-1, a VHL wild type model line of metastatic ccRCC with high VEGF production [35], was maintained in McCoy’s 5A media (ATCC). All media were supplemented with 10% fetal bovine serum, penicillin/streptomycin, and L-glutamine.

### 2.4. Prediction of miRNA Targets

A target search for predicted protein targets was conducted for let-7c-5p using a combination (accessed on 5 May 2021) of TargetScan (http://www.targetscan.org/) [21], miRmap (https://mirmap.ezlab.org/) [22], miRDB (http://mirdb.org/) [23,24], and miRcode (http://www.mircode.org/) [25] analyses. We chose a target gene based on predicted interaction across all search engines with let-7c-5p.

### 2.5. Cell Transfection of pre-miR Constructs

A hemocytometer (American Optical Corporation, Buffalo, NY, USA) was used to count cells and seed 786-O and Caki-1 into CELLSTAR six-well dishes (Greiner, Kremsmunster, Austria) at a density of 2 × 10^4^ cells/mL. siPORT NeoFX transfection reagent (Invitrogen, Carlsbad, CA, USA) was used to deliver 2 μM pre-let-7c-5p (Cat. #AM17100, Assay ID PM10235, Ambion, Austin, TX, USA) or 2 μM pre-miR-Precursor Negative Control #1 (Cat. #AM17110, Ambion) at a final concentration of 20 nM to cells according to the manufacturer’s protocol. The Qiagen miRNeasy mini kit (Qiagen) was used to harvest RNA from transfected cells, and the Epoch spectrophotometer (BioTek, Winooski, VT, USA) was used to evaluate quality and purity (OD 260/280 ratio). Overexpression of let-7c-5p compared to control, RNU43, was conducted using Taqman miRNA qRT-PCR assays according to the manufacturer’s protocol (assay ID: 000379 and 001095, respectively, Applied Biosystems, Foster City, CA, USA). The comparative C_T_ method was used to normalize let-7c-5p expression to RNU43 [36].

### 2.6. Cell Migration and Invasion Assays

In vitro migration and invasion assays were conducted using 24 well plates containing Transwell (8 μm pores; Corning Costar Corp., Cambridge, MA, USA) membrane filter inserts 48 h after transfection. For the migration assay, chambers were placed in wells filled with serum-free medium supplemented with fibronectin (10 μg/mL). Cells (1 × 10^5^ cells/mL) were added to the upper surface of the membrane and allowed to migrate through the membrane for 20 h at 37 °C. The invasion assay followed a similar protocol, albeit with the upper surface of the insert coated with 1:80 Matrigel (Becton-Dickinson, Franklin Lakes, NJ, USA) in serum free media. The inserts were allowed to rest in individual wells containing 10% RPMI for 786-O and 20% McCoy’s for Caki-1 cells. Caki-1 (5 × 10^5^ cells/mL) or 786-O (1 × 10^5^ cells/mL) cells were added to each Transwell chamber and allowed to invade through the membrane for 30 h at 37 °C. For each assay, cells that traversed the membrane were fixed in 10% w/v neutral-buffered formalin (Simport, Quebec, QC, Canada) and stained with DAPI (Invitrogen) (1:500 dilution in PBS, 1% Triton X-100). Cells were visualized using the EVOS FL microscope (Advanced Microscopy Group, Bothwell, WA, USA) and cell counts were recorded from three unique frames captured from each transwell, and a total of three transwells per condition.

### 2.7. Cell Proliferation Assay

Cells were transfected with either pre-let-7c-5p or pre-miR-Precursor Negative Control #1 (NC) and were seeded into 35 mm dishes (Corning) at a density of 2 × 10^4^ cells/mL, in duplicate wells for each. RealTime-Glo™ MT Cell Viability Assay (Promega, Madison, WI, USA) reagents were added at a 1:2000 dilution 48 h after transfection. One hour after the addition of the assay reagents, luminescence readings were obtained on the GloMax^®^ 20/20 Luminometer (Promega). This process was repeated 72 h after transfection.

### 2.8. Western Blot Analysis

Cell lysates were prepared from dishes displaying 70–80% confluency 48 h after transfection. Cells were lysed in 100 μL/well of boiled 1× SDS-Laemmli (250 mM Tris-HCl, 4% SDS, 10% glycerol, 0.003% bromophenol blue) by scraping the dishes manually, followed by shearing with a 24 gauge needle. A BCA assay (Pierce, Waltham, MA, USA) determined the total protein concentration of each sample. Paired lysates from NC and let-7c-5p cells were standardized for total protein concentration and volume and loaded in a unique well of a 12–230 kDa separation module on the Simple Western Jess system (ProteinSimple, Santa Clara, CA, USA). IGF1R primary antibody (NBP1-77679, Novus Biologicals, Littleton, CO, USA) at a concentration of 1:25 diluted in Antibody diluent 2 (ProteinSimple) and rabbit secondary antibody (042-206, ProteinSimple, used as provided) were added according to the manufacturer’s protocol. IGF1R values were normalized against total protein concentration and determined in the same capillary using RePlex and Total Protein Detection reagents (ProteinSimple).

### 2.9. Luciferase Assay

Due to the large size of the 3′UTR of human IGF1R (7115 bp), analysis was performed on three distinct constructs comprising its full length (see Supplemental Materials). The constructs were inserted into vectors linked to the Firefly luciferase gene in addition to the Renilla luciferase gene for normalization (Genecopoeia, Rockville, MD, USA). Each fragment of the IGF1R 3′UTR contained a unique theoretical interaction site between let-7c-5p and the IGF1R gene. Additional mutant constructs were created through site-directed mutagenesis to assess each potential binding site. Caki-1 cells were grown in CELLSTAR 24-well dishes (Greiner) and, upon displaying 70–80% confluency, cell transfection was performed. At this time, the medium was replaced with Opti-MEM™ Reduced Serum Medium (31985062, Invitrogen), and transfected with a vector (0.2 μg) along with either pre-let-7c-5p or pre-miR-Precursor Negative Control #1 (30 nM), delivered with Endofectin (Genecopoeia). Cells were lysed according to the manufacturer’s protocol 24 h following transfection. Firefly and Renilla luminescence was measured using the Luc-Pair™ Duo-Luciferase Assay kit (Genecopoeia) in the GloMax^®^ 20/20 Luminometer (Promega).

### 2.10. Data Analysis

Categorical clinical variables were analyzed using Fisher’s exact tests, and the continuous clinical variables were analyzed using a two-tailed Welch’s *t*-test (SPSS v26). For the miRNA screening analysis, expression levels were normalized to the global mean of each sample for all miRNAs with a Cq < 35 for all samples. Expression levels were quantified as x = 2^(−ΔCt) and log2 transformed. Two-sided Welch’s *t*-tests were performed on the transformed values. Significant differences in miRNA expression levels were determined after adjusting for multiple comparisons using the Benjamini and Hochberg False Discovery Rate (FDR) method: presented as *q*-values. Hierarchical clustering was performed using GENE-E (Broad) with mean-centered values. For cell culture assays, a two-tailed Welch’s *t*-test was conducted to determine whether a statistically significant difference in traits exists for cells undergoing treatment compared to negative control. Corresponding plots depict the mean relative response rate for treated cells relative to negative control. Error bars represent the standard error of the mean. A *p*-value < 0.05 was considered statistically significant. All assays were performed with at least four separate experiments for each cell line.

KEGG (Kyoto Encyclopedia of Genes and Genomes) analysis [37] was performed using DIANA-mirPath v. 3.0 [38]. The analysis was performed using the differentially expressed miRNAs of the let-7 family identified with a *p*-value < 0.05 and a *q*-value (FDR) < 0.25. A heat map was generated with the significant clusters resulting from pathways union analysis using FDR correction with a modified Fisher’s Exact Test, *p* < 0.05. Predicted gene targets with significant enrichment for these miRNAs were based on the miRNA interactions derived from microT-CDS with a threshold setting of 0.8 [39].

## 3. Results

### 3.1. Patient Demographics

Clinical and pathological characteristics related to the tumor samples used in this study are detailed in Table 1. Patients were matched for age and tumor grade and, accordingly, no significant differences were noted for these selected criteria. In addition, no significant difference between the cohorts was observed for tumor size: mean of 3.7 cm for pT1 and 4.1 cm for pT3 tumors.

### 3.2. miRNA Screening Results

**Figure 1:**
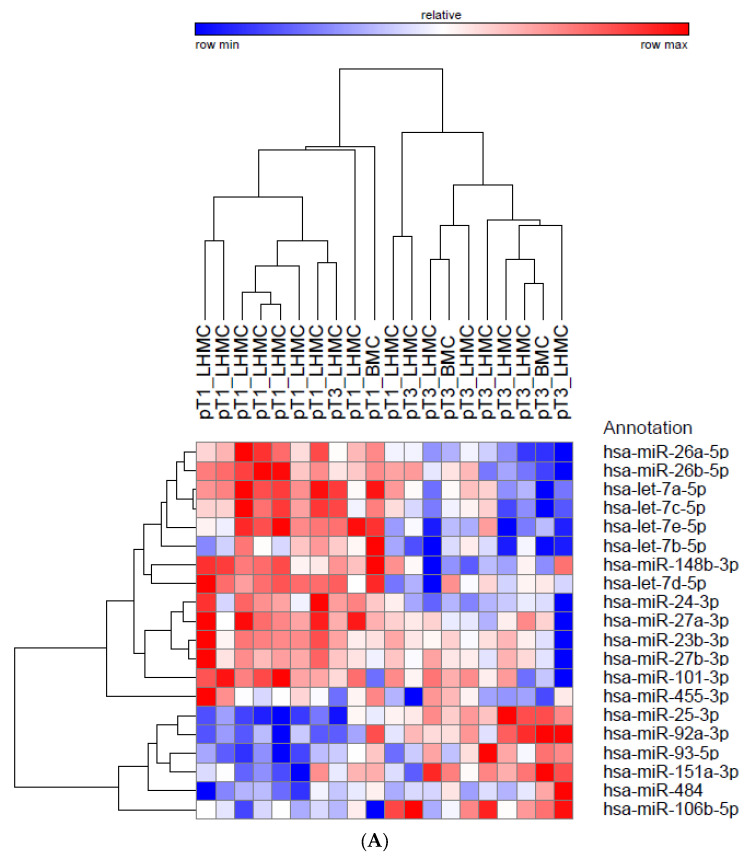

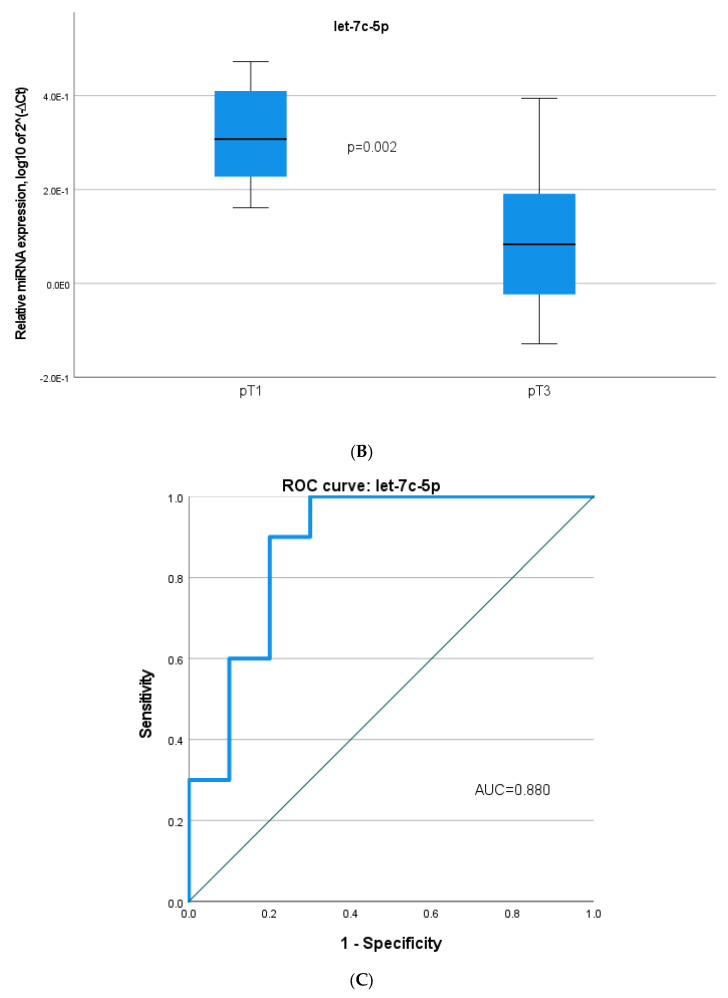

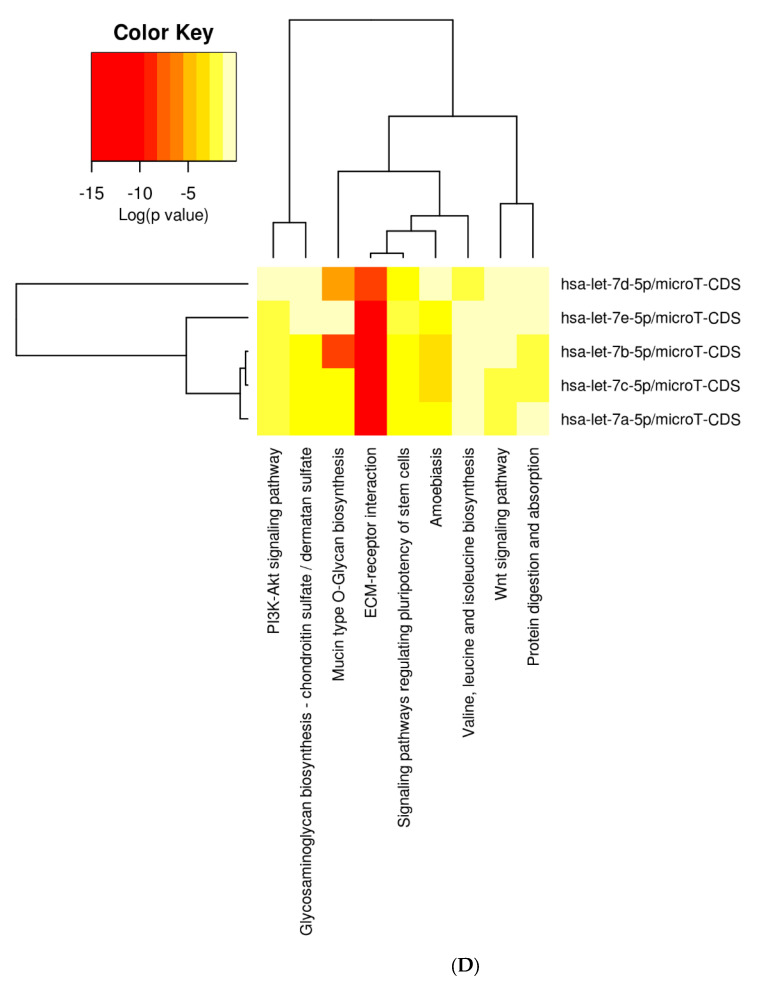
(**A**) Hierarchical clustering analysis utilizing the 20 samples of the screening cohort with the 20 miRNA detected across all samples and differentially expressed (*p* < 0.05 and FDR < 0.25) between pT1 and pT3 tumors (all ≤ 5 cm). (**B**) Relative expression level of let-7c-5p in these specimens. (**C**) ROC curve for let-7c-5p discriminating these pT1 and pT3 cohorts. (**D**) Two-way hierarchical clustering of the significant KEGG pathways, resulting from the analysis of the differentially expressed let-7 miRNAs in this study.

Of the 752 miRNAs analyzed, 108 miRNAs were detected for all samples (Ct < 35). Twenty miRNAs were found to be differentially expressed between the pT1 and pT3 small renal masses (*p* < 0.05 and FDR(q) < 0.25, Table 2). Hierarchical clustering analysis resulted in two distinct clusters differentiating these groups (Figure 1A). Of these 20 miRNAs, seven were upregulated and 13 were downregulated in pT3, compared to the pT1 lesions. Ten of these miRNA had a FDR < 0.05: let-7a-5p, -7c-5p, -7e-5p, miR-24-3p, 25-3p, -26a-5p, -26b-5p, -27a-3p, -93-5p, and -148b-3p. Specifically, let-7c-5p was downregulated 1.6 fold (*p* = 0.0023, FDR = 0.0393, Figure 1B) in this cohort of pT3 tumors, and let-7c-5p discriminated between these groups with an AUC of 0.880 (95%CI 0.721-1.0, Figure 1C).

Five of these dysregulated miRNA are members of the let-7 family: let-7a-5p, -7b-5p, -7c-5p, -7d-5p, and -7e-5p. The KEGG analysis of these miRNAs identified nine predicted pathways (Figure 1D). Many of these pathways are seemingly related to an invasive phenotype. The most significant pathway identified was “ECM receptor interaction” (Supplemental Materials). In addition, 698 predicted genes were identified as being potential targets of at least two members of the let-7 family, and all five were predicted to target IGF1R (Supplemental Materials).

### 3.3. Transfection of 786-0 and Caki-1 Cells with let-7c-5p Inhibits Proliferation, Migration, and Invasion In Vitro

**Figure 2:**
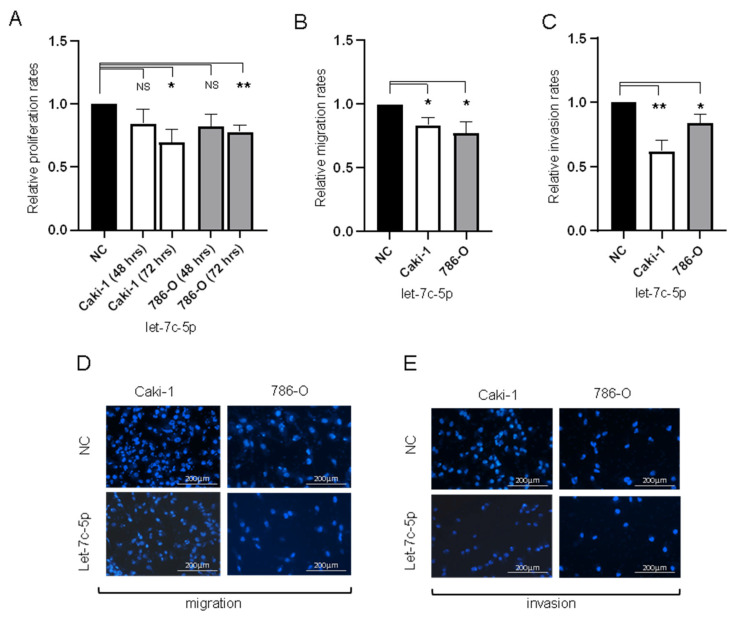
Influence of let-7c-5p transfection on cell behavior in Caki-1 and 786-O cells (**A**) Relative proliferation rates of Caki-1 and 786-O cells transfected with pre-let-7c-5p compared to scramble negative control. (**B**) Relative migration rates of Caki-1 and 786-O cells transfected with pre-let-7c-5p compared to negative control transfectants. (**C**) Relative invasion rates of Caki-1 and 786-O cells transfected with pre-let-7c-5p compared to negative control. (**D**) representative images from the migration assay. (**E**) representative images from the invasion assay. In each case, the relative rates were decreased in cells treated with the let-7c-5p mimic. * *p* < 0.05, ** *p* < 0.01, and NS = not significant.

Let-7c-5p expression was rescued in 786-O and Caki-1 cell lines with pre-let-7c-5p and compared with a scrambled sequence as a negative control (Supplemental Materials). Forty-eight hours post-transfection, cells were tested in the Promega Cell Viability Assay to assess proliferation. While transfection with let-7c-5p did not significantly alter the proliferation of 786-O and Caki-1 cells after 48 h, at 72 h, each cell line displayed significantly diminished proliferation when transfected with pre-let-7c-5p as opposed to negative control (Figure 2A). Cell migration and invasion assays were conducted to explore the role of let-7c-5p in the EMT phenotype. Transfection of 786-O and Caki-1 cells with pre-let-7c-5p compared to negative control significantly reduced both cell migration and invasion in each cell line (Figure 2B,C). The net effect of increasing let-7c-5p levels in these cells resulted in phenotypic changes consistent with reduced metastatic potential.

### 3.4. IGF1R Expression Is Directly Regulated by let-7c-5p

**Figure 3:**
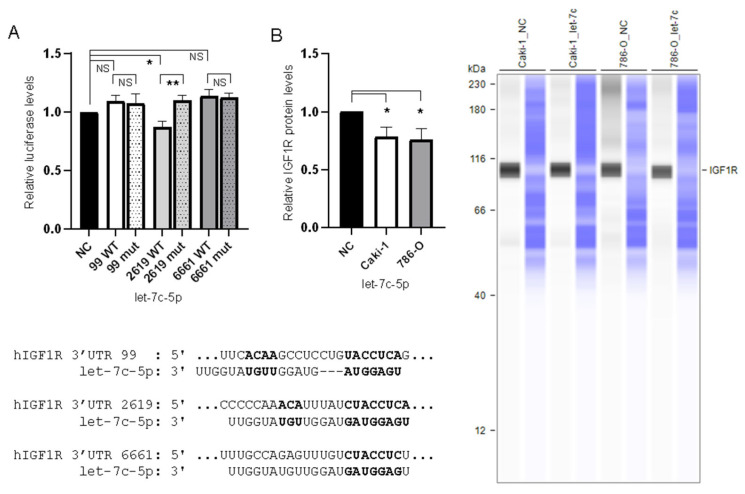
The effect of let-7c-5p on the expression of IGF1R. (**A**) Relative luciferase activity of Caki-1 cells for each vector, including mutants, transfected with pre-let-7c-5p, relative to the negative control transfectant (predicted sites detailed in the inset below the chart). (**B**) Relative expression of IGF1R in Caki-1 and 786-O cells transfected with let-7c-5p after 48 h, relative to the negative control for each cell line. Representative image inset to the right: the first lane of each pair corresponds to IGF1R and the second is total protein detected for the same lane. * *p* < 0.05, ** *p* < 0.01, and NS = not significant.

IGF1R was identified as a direct target of let-7c-5p by each of the prediction algorithms used in this study [21,22,23,24,25], and our analysis focused on the three sites consistently identified by all of these prediction algorithms. These sites are referred to as 99, 2619, and 6661, based on their location in the 3′UTR of IGF1R (see Figure 3A and Supplemental Material). To confirm the direct interaction between let-7c-5p and the 3′UTR of IGF1R, a dual luciferase reporter assay was conducted in Caki-1 cells (Figure 3A). Unique vectors were constructed, each comprising ~2400 bp of the 3′UTR of IGF1R (see Supplemental Material for details), with each site present on a distinctive construct. Relative luciferase activity was calculated for each group through the Renilla/Firefly luminescence ratio. A significant reduction in relative luciferase activity was observed following co-transfection with let-7c-5p and the 2619 plasmid compared to the negative control and the 2619 mutant plasmid. Conversely, no significant difference was detected between cells co-transfected with let-7c-5p and the mutant 2619 plasmid compared to the negative control. When let-7c-5p was transfected with either the wild-type (WT) 99 or 6661 vector, and their mutant versions, no significant change was noted for WT compared to negative control or mutant. Western blot analysis, using the Jess Simple Western System, was conducted to further assess the impact of let-7c-5p on IGF1R expression levels in ccRCC cells. Transfection of 786-O and Caki-1 cells with pre-let-7c-5p as opposed to a negative control pre-miRNA construct significantly decreased IGF1R protein expression (Figure 3B).

## 4. Discussion

Recent studies have focused on the prognostic ability of miRNA expression signatures to predict patient outcomes and their influence on carcinogenesis. miRNA dysregulation drives altered protein expression, namely through tumor suppressors and oncogenes. A previous study from our laboratory investigated pT1 tumors, identifying a number of miRNA as dysregulated in tumors that progressed to metastatic disease [6]. The present study further investigated the role of miRNA expression in the metastatic phenotype of small ccRCC tumors. Comparing cT1 tumors classified as pT1 with those pathologically upstaged to pT3a at the time of surgery elucidated a number of dysregulated miRNA that may play a role in conferring invasive properties within the tumor. One miRNA that has been downregulated in both studies, within aggressive pT1 tumors as well as cT1 tumors that have been upstaged to pT3a at the time of surgery, is let-7c-5p.

The present study further demonstrated the role of let-7c-5p expression in the metastatic phenotype in ccRCC cells. Caki-1 and 786-O cells transfected with pre-let-7c-5p displayed a markedly reduced rate of proliferation, migration, and invasion compared to cells transfected with a negative control construct. We also confirm the interaction between let-7c-5p and IGF1R, an oncogene transmembrane receptor that displays tyrosine kinase activity when bound to IGF-1 [26], in ccRCC cells. Through luciferase and western blot analysis, we verify that let-7c-5p decreases IGF1R expression by binding to its 3′UTR. We hypothesize that reduced expression of let-7c-5p contributes to the overexpression of IGF1R in ccRCC cells, which contributes to tumor progression and metastasis.

Interestingly, four other members of the let-7 family were identified as being dysregulated in this study (let -7a, -b, -d, and -e). These miRNAs share the same seed sequence (GAUGGAG) as let-7c-5p that is complimentary to the 3′UTR of IGF1R. Conceivably, one or more of these other family members may also be targeting IGF1R in ccRCC and therefore may cooperate in an additive manner to enhance regulation of IGF1R—as well as potentially other oncogenes. KEGG analysis of these miRNAs predicted their involvement in pathways related to the invasive phenotype, such as PI3K-AKT signaling, Wnt signaling, and the most significant process in our analysis—ECM receptor interaction. PI3K-AKT and Wnt signaling pathways have been well studied in oncogenesis [40], and they have been implicated specifically in the invasive phenotype of ccRCC [41,42]. An examination of the ECM receptor interaction pathway (Supplemental Materials) identified proteins directly of interest to this study. Various collagens and integrins, most notably, are members of this pathway that are likely targeted by multiple members of the let-7 family dysregulated in this study (Supplemental Material).

Integrins are cell surface molecules that are involved in cell–cell and cell extracellular matrix adhesion and play important roles in cell proliferation, migration, and signaling [43]. Cross-talk between integrins and the IGF1R signaling pathway has been well documented [44,45]. Additionally, intergrin expression levels have been correlated with patient survival and metastasis, which suggests that some integrins may play a vital role in cancer progression. Specific to renal cell carcinoma, Breuksch et al. reported that the integrin α5 expression correlated with distant metastases within five years after tumor nephrectomy and reduced survival [46]. Collagens and other associated ECM components also have the potential to influence IGF1R signaling [47]. Collagen levels have been correlated with cancer invasion and lymph node metastasis [48,49,50,51] and have been linked to poor prognosis in several cancers, including ccRCC [52,53]. The concept that ECM ligands bind to integrins and IGF1 binds to IGF1R has been proposed [54]. The let-7 family as well as some of its targets, including integrins and collagen, have all been associated with invasive and metastatic properties and linked to poor prognosis in ccRCC. Collectively, this evidence supports the role of the let-7 family as agents of tumor suppression in small renal masses. Thus, it is interesting to speculate on the therapeutic potential of inhibiting ccRCC tumors by systemic delivery of let-7 mimics, as demonstrated in a mouse model of lung cancer [55]. A limitation of this study is that we only investigated the binding of let-7c-5p in this work. It was the let family member that was most consistently dysregulated across this study and our previous investigation of aggressive small renal masses [6], and it is arguably the most noted let-7 family member reported as being dysregulated in ccRCC.

Studies on let-7c-5p have demonstrated the miRNA to be downregulated in ccRCC as well as other cancer types. Peng et al. found let-7c-5p to be downregulated in ccRCC tissue compared to adjacent normal, and correlated the downregulation with increased chemoresistance to the drug 5-fluorouracil [11]. Additionally, let-7c-5p expression has been shown to be significantly decreased in early metastatic ccRCC tumors compared with non-metastatic tumors [9]. Low expression of let-7c-5p has been correlated with disease state in a variety of other cancers, such as prostate [13,14], acute promyelocytic leukemia [15], hepatocellular [16], non-small cell lung [17], and endometrial [18]. Specifically, in non-small cell lung cancer, Zhao et al. detected a significant association between let-7c-5p downregulation, venous invasion, and metastasis [17], which is consistent with our results within small ccRCC tumors. Conversely, while low let-7c-5p expression is frequently correlated with poor patient outcomes in human cancers, there is some evidence of let-7c-5p acting as an oncogene. High expression levels have been found in aggressive cancers with poorer patient prognosis in high grade serous ovarian carcinoma [56] and oral tongue squamous cell carcinoma [57].

While let-7c-5p undoubtedly targets the 3′UTR of many protein transcripts, we chose to focus on IGF1R because of its predicted interaction with let-7c-5p and its association with poorer prognosis for ccRCC patients. The link between these two factors has previously been established in head and neck squamous cell carcinoma via a luciferase reporter assay [27] but, to our knowledge, has not been investigated in ccRCC. IGF1R is a transmembrane receptor that exerts its influence on cell behavior through the activation of various downstream effectors such as the PI3K-AKT network, mitogen activated protein kinase (MAPK) pathway, and mTOR [58]. Expression levels of IGF1R correlate with Fuhrman grading in ccRCC tissues [33], and high IGF1R expression confers a significant reduction in cancer specific survival [32]. High expression of IGF1R has also been shown to induce clathrin-dependent endocytosis in which IGF1R is translocated to the nucleus, which is associated with adverse prognosis [59]. Heightened expression of IGF1R has also been identified within ccRCC biopsy compared to normal kidney, and inactivation of the von Hippel–Lindau tumor suppressor gene has been directly linked to this upregulation [60]. In ccRCC cells, siRNA-induced knockdown of IGF1R leads to diminished migratory and invasive abilities, along with a reduction in proliferation [30,31]. Additionally, Yuen et al. demonstrated the effects of IGF1R depletion on chemoresistance, as ccRCC cells became desensitized to 5-fluorouracil and etoposide [58]. Interestingly, both let-7c depletion and IGF1R overexpression have been shown to increase chemoresistance to 5-fluorouracil. These findings together suggest a potential therapeutic benefit from abrogating the dysregulation of these two factors.

While we chose to focus on the role of let-7c-5p in the invasive capacity of small ccRCC tumors, other miRNA that have been implicated both in the present study as well as prior work from our laboratory on pT1 ccRCC. miR-25-3p, miR-93-5p, and miR-484 have all been identified as upregulated in aggressive ccRCC tumors, while miR-26a-5p, 26b-5p, miR-23b-3p, miR-27a-3p, miR-148b-3p, let-7e-5p, and let-7b-5p expression levels are reduced. Evaluation of the influence of these miRNA on ccRCC cell behavior and the protein targets that they regulate may improve our understanding of why these small tumors progress to metastatic disease.

## 5. Conclusions

In this study, we identify a subset of miRNA that is dysregulated in small (<5 cm) cT1 ccRCC that were later upstaged to pT3 at the time of surgery due to the presence of invasion. Members of the let-7c family were downregulated in these tumors, as well as in pT1 tumors that would later progress to metastatic disease. Dysregulation of these miRNAs facilitates the metastatic phenotype and portends a poor prognosis. The reduced expression of the let-7 family likely contributes to the upregulation of IGF1R in these tumors, and we demonstrate that let-7c-5p modulates proliferation, migration, invasion, and IGF1R expression in ccRCC cells.

## Figures and Tables

**Table 1:** Clinical and pathological characteristics for all of the samples in this study.

Variable	pT1	pT3	*p*-Value
Number of samples	10	10	
Age at surgery, years, mean (range)	63 (43–76)	63 (43–78)	0.912
Gender, n (%)			0.628
Female	4 (40)	2 (20)	
Male	6 (60)	8 (80)	
Grade, n (%)			1
2	4 (40)	3 (30)	
3	4 (40)	4 (40)	
4	2 (20)	3 (30)	
tumor size, cm, median (range)	3.7 (2–5)	4.1 (2–5)	0.373

**Table 2:** The median fold change (FC) in miRNA expression between the samples in this study. A negative fold change indicates lower expression in pT3 tumors.

microRNA	FC	*p*-Value	*q*-Value
**hsa-miR-26a-5p**	−1.9	<0.001	0.005
**hsa-miR-26b-5p**	−1.82	<0.001	0.024
hsa-miR-24-3p	−1.42	0.001	0.024
**hsa-miR-25-3p**	1.84	0.001	0.024
hsa-let-7a-5p	−1.65	0.001	0.024
**hsa-miR-148b-3p**	−1.61	0.001	0.024
**hsa-miR-93-5p**	1.32	0.001	0.024
**hsa-let-7e-5p**	−1.58	0.002	0.038
**hsa-let-7c-5p**	−1.58	0.002	0.039
**hsa-miR-27a-3p**	−1.5	0.003	0.048
hsa-miR-92a-3p	1.5	0.004	0.053
**hsa-miR-23b-3p**	−1.5	0.013	0.154
**hsa-let-7b-5p**	−1.47	0.015	0.154
hsa-miR-151a-3p	1.49	0.018	0.166
hsa-let-7d-5p	−1.31	0.022	0.188
hsa-miR-106b-5p	1.27	0.025	0.201
**hsa-miR-484**	1.65	0.033	0.227
hsa-miR-101-3p	−1.53	0.033	0.227
hsa-miR-455-3p	−1.7	0.036	0.235
hsa-miR-27b-3p	−1.45	0.038	0.242

Bold face indicates miRNA that were also characteristic of aggressive pT1 lesions in a previous study from our laboratory.

## Data Availability

The data presented in this study are available on request from the corresponding author.

## Supplementary Materials

The following supporting information can be downloaded at: https://www.mdpi.com/article/10.3390/biomedicines10102425/s1, sequence for the three constructs comprising the full-length 3′UTR of IGF1R; Relative let-7c-5p expression in transfectants relative to corresponding negative controls.
