# Supplementary material for: MicroRNA Associated with the Invasive Phenotype in Clear Cell Renal Cell Carcinoma: Let-7c-5p Inhibits Proliferation, Migration, and Invasion by Targeting Insulin-like Growth Factor 1 Receptor
Source: Biomedicines. 2022 Sep 28;10(10):2425. doi: 10.3390/biomedicines10102425 (PMC9598558; doi:10.3390/biomedicines10102425)
Supplement: Supplementary file 1 [file biomedicines-10-02425-s001.zip › biomedicines-1906932-supplementary.pdf]

Supplemental Material

Wild-type sequence for the three constructs comprising the full-length 3'UTR of IGF1R that were utilized in this study. The three mutated sites are highlighted in blue, and the mutated sequence is detailed in the header preceding each construct.

>HmiT100884a: sites 99-105 changed from tacctca to atggagt
GATCCTTGGATCCTGAATCTGTGCAAACAGTAACGTGTGCGCACGCGCAGCGGGGTGGGGGGGAGAGAGA
GTTTTAACAATCCATTCACAAGCCTCCTGTACCTCAGTGGATCTTCAGAACTGCCCTTGCTGCCCGCGGGAGA
CAGCTTCTCTGCAGTAAAACACATTTGGGATGTTCCTTTTTTCAATATGCAAGCAGCTTTTTATTCCCTGCCCA
AACCCTTAACTGACATGGGCCTTTAAGAACCTTAATGACAACACTTAATAGCAACAGAGCACTTGAGAACC
AGTCTCCTCACTCTGTCCCTGTCCTTCCCTGTTCTCCCTTTCTCTCTCCTCTCTGCTTCATAACGGAAAAATAAT
TGCCACAAGTCCAGCTGGGAAGCCCTTTTTATCAGTTTGAGGAAGTGGCTGTCCCTGTGGCCCCATCCAACC
ACTGTACACACCCGCCTGACACCGTGGGTCATTACAAAAAAACACGTGGAGATGGAAATTTTTACCTTTATC
TTTCACCTTTCTAGGGACATGAAATTTACAAAGGGCCATCGTTCATCCAAGGCTGTTACCATTTTAACGCTGC
CTAATTTTGCCAAAATCCTGAACTTTCTCCCTCATCGGCCCGGCGCTGATTCCTCGTGTCCGGAGGCATGGGT
GAGCATGGCAGCTGGTTGCTCCATTTGAGAGACACGCTGGCGACACACTCCGTCCATCCGACTGCCCCTGCT
GTGCTGCTCAAGGCCACAGGCACACAGGTCTCATTGCTTCTGACTAGATTATTATTTGGGGGAACTGGACAC
AATAGGTCTTTCTCTCAGTGAAGGTGGGGAGAAGCTGAACCGGCTTCCCTGCCCTGCCTCCCCAGCCCCCTG
CCCAACCCCCAAGAATCTGGTGGCCATGGGCCCCGAAGCAGCCTGGCGGACAGGCTTGGAGTCAAGGGGCC
CCATGCCTGCTTCTCTCCCAGCCCCAGCTCCCCCGCCCGCCCCCAAGGACACAGATGGGAAGGGGTTTCCAG
GGACTCAGCCCCACTGTTGATGCAGGTTTGCAAGGAAAGAAATTCAAACACCACAACAGCAGTAAGAAGA
AAAGCAGTCAATGGATTCAAGCATTCTAAGCTTTGTTGACATTTTCTCTGTTCCTAGGACTTCTTCATGGGTCT
TACAGTTCTATGTTAGACCATGAAACATTTGCATACACATCGTCTTTAATGTCACTTTTATAACTTTTTTACGG
TTCAGATATTCATCTATACGTCTGTACAGAAAAAAAAAAAGCTGCTATTTTTTTTGTTCTTGATCTTTGTGGATT
TAATCTATGAAAACCTTCAGGTCCACCCTCTCCCCTTTCTGCTCACTCCAAGAAACTTCTTATGCTTTGTACTA
GAGTGCGTGACTTTCTTCCTCTTTTCCCGGTAATGGATACTTCTATCACATAATTTGCCATGAACTGTTGGATG
CCTTTTTATAAATACATCCCCCATCCCTGCTCCCACCTGCCCCTTTAGTTGTTTTCTAACCCGTAGGCTCTCTGG
GCACGAGGCAGAAAGCAGGCCGGGCACCCATCCTGAGAGGGCCGCGCTCCTCTCCCCAGCCTGCCCTCACA
GCATTGGAGCCTGTTACAGTGCAAGACATGATACAAACTCAGGTCAGAAAAACAAAGGTTAAATATTTCAC
ACGTCTTTGTTCAGTGTTTCCACTCACCGTGGTTGAGAAGCCTCACCCTCTCTTTCCCTTGCCTTTGCTTAGGTT
GTGACACACATATATATATATTTTTTTAATTCTTGGGTACAACAGCAGTGTTAACCGCAGACACTAGGCATTT
GGATTACTATTTTTCTTAATGGCTATTTAATCCTTCCATCCCACGAAAAACAGCTGCTGAGTCCAAGGGAGCA
GCAGAGCGTGGTCCGGCAGGGCCTGTTGTGGCCCTCGCCACCCCCCTCACCGGACCGACTGACCTGTCTTTG
GAACCAGAACATCCCAAGGGAACTCCTTCGCACTGGCGTTGAGTGGGACCCCGGGATCCAGGCTGGCCCAG
GGCGGCACCCTCAGGGCTGTGCCCGCTGGAGTGCTAGGTGGAGGCAGCACAGACGCCACGGTGGCCCAAGA
GCCCCTTTGCTTCTTGCTGGGGGACCAGGGCTGTGGTGCTGGCCCACTTTCCCTCGGCCAGGAATCCAGGTCC
TTGGGGCCCAGGGGTCTTGTCTTGTTTCATTTTTAGCACTTCTCACCAGAGAGATGACAGCACAAGAGTTGCT
TCTGGGATAGAAATGTTTAGGAGTAAGAACAAAGCTGGGATACGGTGATTGCTAGTTGTGACTGAAGATTCA
ACACAGAAAAGAAAGTTTATACGGCTTTTTTGCTGGTCAGCAGTTTGTCCCACTGCTTTCTCTAGTCTCTATCC
CATAGCGTGTTCCCTTTAAAAAAAAAAAAAAAGGTATTATATGTAGGAGTTTTCTTTTAATTTATTTTGTGATA
AATTACCAGTTTCAATCACTGTAGAAAAGCCCCATTATGAATTTAAATTTCAAGGAAAGGGTGTGTGTGTGT
GTATGTGTGGGGTGTGTGTGTGTGAGAGTGATGGGACAGTTCTTGATT

>HmiT100884b: sites 2619-2626 changed from ctacctca to gatggagt
CAATCACTGTAGAAAAGCCCCATTATGAATTTAAATTTCAAGGAAAGGGTGTGTGTGTGTGTATGTGTGGGG
TGTGTGTGTGTGAGAGTGATGGGACAGTTCTTGATTTTTTGGGTTTTTTTTCCCCCAAACATTTATCTACCTCAC
TCTTATTTTTTATATGTGTATATAGACAAAAGAATACATCTCACCTTTCTCAGCACCTGACAATAGGCCGTTG
ATACTGGTAACCTCATCCACGCCACAGGCGCCACACCCAGGTGATGCAGGGGGAAGCCAGGCTGTATTCCG
GGGTCAAAGCAACACTAACTCACCTCTCTGCTCATTTCAGACAGCTTGCCTTTTTCTGAGATGTCCTGTTTTGT

GTTGCTTTTTTTGTTTTGTTTTCTATCTTGGTTTCCACCAAGGTGTTAGATTTCTCCTCCTCCTAGCCAGGTGGCC
CTGTGAGGCCAACGAGGGCACCAGAGCACACCTGGGGGAGCCACCAGGCTGTCCCTGGCTGGTTGTCTTTG
GAACAAACTGCTTCTGTGCAGATGGAATGACCAACACATTTCGTCCTTAAGAGAGCAGTGGTTCCTCAGGTT
CTGAGGAGAGGAAGGTGTCCAGGCAGCACCATCTCTGTGCGAATCCCCAGGGTAAAGGCGTGGGGCATTGG
GTTTGCTCCCCTTGCTGCTGCTCCATCCCTGCAGGAGGCTCGCGCTGAGGCAGGACCGTGCGGCCATGGCTGC
TGCATTCATTGAGCACAAAGGTGCAGCTGCAGCAGCAGCTGGAGAGCAAGAGTCACCCAGCCTGTGCGCCA
GAATGCAGAGGCTCCTGACCTCACAGCCAGTCCCTGATAGAACACACGCAGGAGCAGAGTCCCCTCCCCCT
CCAGGCTGCCCTCTCAACTTCTCCCTCACCTCCTTCCCTAGGGGTAGACAGAGATGTACCAAACCTTCCGGCT
GGAAAGCCCAGTGGCCGGCGCCGAGGCTCGTGGCGTCACGCCCCCCCCGCCAGGGCTGTACCTCCGTCTCCC
TGGTCCTGCTGCTCACAGGACAGACGGCTCGCTCCCCTCTTCCAGCAGCTGCTCTTACAGGCACTGATGATTT
CGCTGGGAAGTGTGGCGGGCAGCTTTGCCTAAGCGTGGATGGCTCCTCGGCAATTCCAGCCTAAGTGAAGGC
GCTCAGGAGCCTCCTGCTGGAACGCGACCCATCTCTCCCAGGACCCCGGGGATCTTAAGGTCATTGAGAAAT
ACTGTTGGATCAGGGTTTTGTTCTTCCACACTGTAGGTGACCCCTTGGAATAACGGCCTCTCCTCTCGTGCAC
ATACCTACCGGTTTCCACAACTGGATTTCTACAGATCATTCAGCTGGTTATAAGGGTTTTGTTTAAACTGTCCG
AGTTACTGATGTCATTTTGTTTTTGTTTTATGTAGGTAGCTTTTAAGTAGAAAACACTAACAGTGTAGTGCCCA
TCATAGCAAATGCTTCAGAAACACCTCAATAAAAGAGAAAACTTGGCTTGTGTGATGGTGCAGTCACTTTAC
TGGACCAACCCACCCACCTTGACTATACCAAGGCATCATCTATCCACAGTTCTAGCCTAACTTCATGCTGATT
TCTCTGCCTCTTGATTTTTCTCTGTGTGTTCCAAATAATCTTAAGCTGAGTTGTGGCATTTTCCATGCAACCTCC
TTCTGCCAGCAGCTCACACTGCTTGAAGTCATATGAACCACTGAGGCACATCATGGAATTGATGTGAGCATT
AAGACGTTCTCCCACACAGCCCTTCCCTGAGGCAGCAGGAGCTGGTGTGTACTGGAGACACTGTTGAACTTG
ATCAAGACCCAGACCACCCCAGGTCTCCTTCGTGGGATGTCATGACGTTTGACATACCTTTGGAACGAGCCT
CCTCCTTGGAAGATGGAAGACCGTGTTCGTGGCCGACCTGGCCTCTCCTGGCCTGTTTCTTAAGATGCGGAGT
CACATTTCAATGGTACGAAAAGTGGCTTCGTAAAATAGAAGAGCAGTCACTGTGGAACTACCAAATGGCGA
GATGCTCGGTGCACATTGGGGTGCTTTGGGATAAAAGATTTATGAGCCAACTATTCTCTGGCACCAGATTCTA
GGCCAGTTTGTTCCACTGAAGCTTTTCCCACAGCAGTCCACCTCTGCAGGCTGGCAGCCGAATGGCTTGCCA
GTGGCTCTGTGGCAAGATCACACTGAGATCGATGGGTGAGAAGGCTAGGATGCTTGTCTAGTGTTCTTAGCT
GTCACGTTGGCTCCTTCCAGGGTGGCCAGACGGTGTTGGCCACTCCCTTCTAAAACACAGGCGCCCTCCTGGT
GACAGTGACCCGCCGTGGTATGCCTTGGCCCATTCCAGCAGTCCCAGTTATGCATTTCAAGTTTGGGGTTTGT
TCT

>HmiT100884c: sites 6661-6667 changed from ctacctc to gatggag
CATTCCAGCAGTCCCAGTTATGCATTTCAAGTTTGGGGTTTGTTCTTTTCGTTAATGTTCCTCTGTGTTGTCAGC
TGTCTTCATTTCCTGGGCTAAGCAGCATTGGGAGATGTGGACCAGAGATCCACTCCTTAAGAACCAGTGGCG
AAAGACACTTTCTTTCTTCACTCTGAAGTAGCTGGTGGTACAAATGAGAACTTCAAGAGAGGATGTTATTTAG
ACTGAACCTCTGTTGCCAGAGATGCTGAAGATACAGACCTTGGACAGGTCAGAGGGTTTCATTTTTGGCCTTC
ATCTTAGATGACTGGTTGCGTCATTTGGAGAAGTGAGTGCTCCTTGATGGTGGAATGACCGGGTGGTGGGTA
CAGAACCATTGTCACAGGGATCCTGGCACAGAGAAGAGTTACGAGCAGCAGGGTGCAGGGCTTGGAAGGA
ATGTGGGCAAGGTTTTGAACTTGATTGTTCTTGAAGCTATCAGACCACATCGAGGCTCAGCAGTCATCCGTGG
GCATTTGGTTTCAACAAAGAAACCTAACATCCTACTCTGGAAACTGATCTCGGAGTTAAGGCGAATTGTTCA
AGAACACAAACTACATCGCACTCGTCAGTTGTCAGTTCTGGGGCATGACTTTAGCGTTTTGTTTCTGCGAGAA
CATAACGATCACTCATTTTTATGTCCCACGTGTGTGTGTCCGCATCTTTCTGGTCAACATTGTTTTAACTAGTC
ACTCATTAGCGTTTTCAATAGGGCTCTTAAGTCCAGTAGATTACGGGTAGTCAGTTGACGAAGATCTGGTTTA
CAAGAACTAATTAAATGTTTCATTGCATTTTTGTAAGAACAGAATAATTTTATAAAATGTTTGTAGTTTATAAT
TGCCGAAAATAATTTAAAGACACTTTTTTTTTCTCTGTGTGTGCAAATGTGTGTTTGTGATCCATTTTTTTTTTTT
TTTTTTAGGACACCTGTTTACTAGCTAGCTTTACAATATGCCAAAAAAGGATTTCTCCCTGACCCCATCCGTG
GTTCACCCTCTTTTCCCCCCATGCTTTTTGCCCTAGTTTATAACAAAGGAATGATGATGATTTAAAAAGTAGTT
CTGTATCTTCAGTATCTTGGTCTTCCAGAACCCTCTGGTTGGGAAGGGGATCATTTTTTACTGGTCATTTCCCTT
TGGAGTGTAGCTACTTTAACAGATGGAAAGAACCTCATTGGCCATGGAAACAGCCGAGGTGTTGGAGCCCA
GCAGTGCATGGCACCGTTCGGCATCTGGCTTGATTGGTCTGGCTGCCGTCATTGTCAGCACAGTGCCATGGAC
ATGGGAAGACTTGACTGCACAGCCAATGGTTTTCATGATGATTACAGCATACACAGTGATCACATAAACGAT

GACAGCTATGGGGCACACAGGCCATTTGCTTACATGCCTCGTATCATGACTGATTACTGCTTTGTTAGAACAC
AGAAGAGACCCTATTTTATTTAAGGCAGAACCCCGAAGATACGTATTTCCAATACAGAAAAGAATTTTTAAT
AAAAACTATAACATACACAAAAATTGGTTTTAAAGTTGACTCCACTTCCTCTAACTCCAGTGGATTGTTGGCC
ATGTCTCCCCAACTCCACAATATCTCTATCATGGGAAACACCTGGGGTTTTTGCGCTACATAGGAGAAAGAT
CTGGAAACTATTTGGGTTTTGTTTTCAACTTTTCATTTGGATGTTTGGCGTTGCACACACACATCCACCGGTGG
AAGAGACGCCCGGTGAAAACACCTGTCTGCTTTCTAAGCCAGTGAGGTTGAGGTGAGAGGTTTGCCAGAGTT
TGTCTACCTCTGGGTATCCCTTTGTCTGGGATAAAAAAAATCAAACCAGAAGGCGGGATGGAATGGATGCAC
CGCAAATAATGCATTTTCTGAGTTTTCTTGTTAAAAAAAAAATTTTTTTAAGTAAGAAAAAAAAAAGGTAATAA
CATGGCCAATTTGTTACATAAAATGACTTTCTGTGTATAAATTATTCCTAAAAAATCCTGTTTATATAAAAAA
TCAGTAGATGAAAAAAATTTCAAAATGTTTTTGTATATTCTGTTGTAAGAATTTATTCCTGTTATTGCGATATA
CTCTGGATTCTTTACATAATGGAAAAAAGAAACTGTCTATTTTGAATGGCTGAAGCTAAGGCAACGTTAGTTT
CTCTTACTCTGCTTTTTTCTAGTAAAGTACTACATGGTTTAAGTTAAATAAAATAATTCTGTATGCATT

Relative let-7c-5p expression in transfectants relative to corresponding negative controls.

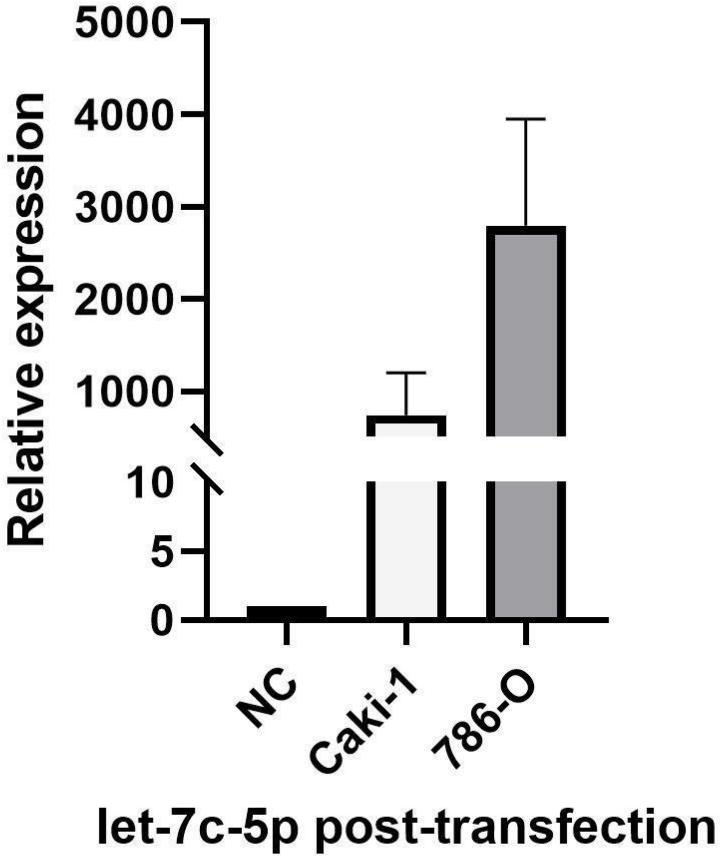

The KEGG pathway ECM-receptor interaction. This pathway facilitates cellular activities such as adhesion, migration, differentiation, proliferation, and apoptosis (https://www.genome.jp/pathway/hsa04512, Kanehisa Lab).

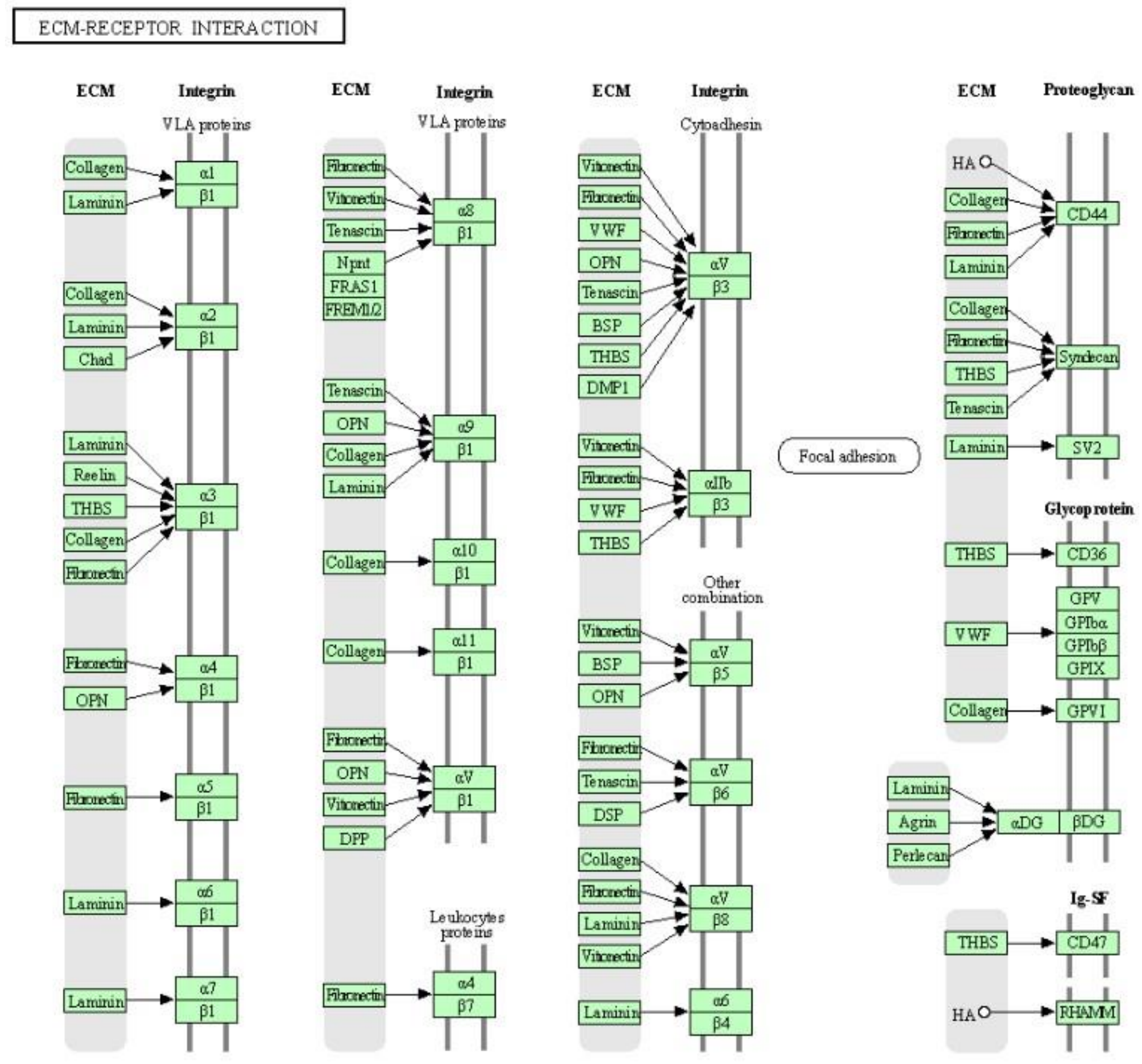

A list of genes targeted by at least two members of the let-7 family in this study (let-7a-5p, -7b-5p, -7c5p, -7d-5p, and -7e-5p). The number of matches refers to the number of unique let-7 family members identified by microT-CDS (threshold 0.8) for each gene.

Gene	Ensembl ID	let-7 matches
ABCC5	ENSG00000114770	5
ABCC8	ENSG00000006071	4
ABCD4	ENSG00000119688	4
ABL2	ENSG00000143322	5
AC004381.6	ENSG00000005189	4
AC023632.1	ENSG00000212997	5

ACP6	ENSG00000162836	2
ACSL6	ENSG00000164398	5
ACTA1	ENSG00000143632	4
ACTB	ENSG00000075624	5
ACTG2	ENSG00000163017	5
ACVR1B	ENSG00000135503	5
ACVR1C	ENSG00000123612	5
ACVR2A	ENSG00000121989	5
ADA	ENSG00000196839	3
ADAMTS12	ENSG00000151388	5
ADAMTS15	ENSG00000166106	5
ADAMTS5	ENSG00000154736	5
ADAMTS8	ENSG00000134917	5
ADAP2	ENSG00000184060	5
ADIPOR2	ENSG00000006831	4
ADRB1	ENSG00000043591	5
ADRB2	ENSG00000169252	5
ADRB3	ENSG00000188778	5
AEN	ENSG00000181026	4
AGAP1	ENSG00000157985	3
AGBL2	ENSG00000165923	4
AGO3	ENSG00000126070	5
AGO4	ENSG00000134698	4
AGPAT6	ENSG00000158669	3
AHCTF1	ENSG00000153207	5
AIFM1	ENSG00000156709	5
AKAP8	ENSG00000105127	4
AL136115.1	ENSG00000212673	4
ALG8	ENSG00000159063	2
ALPK1	ENSG00000073331	4
AMER3	ENSG00000178171	4
ANKRD49	ENSG00000168876	5
AP000708.1	ENSG00000255537	2
AP1S1	ENSG00000106367	2

ARG2	ENSG00000081181	3
ARHGAP12	ENSG00000165322	4
ARHGAP28	ENSG00000088756	5
ARHGAP8	ENSG00000241484	4
ARHGEF15	ENSG00000198844	2
ARHGEF28	ENSG00000214944	4
ARHGEF38	ENSG00000236699	5
ARID3A	ENSG00000116017	5
ARID3B	ENSG00000179361	5
ARIH1	ENSG00000166233	3
ARL6IP6	ENSG00000177917	4
ARPP19	ENSG00000128989	4
ASAP1	ENSG00000153317	4
ASCC3	ENSG00000112249	3
ASIC1	ENSG00000110881	4
ATP2A2	ENSG00000174437	4
ATPAF1	ENSG00000123472	5
B3GNT1	ENSG00000174684	4
B3GNT7	ENSG00000156966	3
BACH1	ENSG00000156273	5
BCAP29	ENSG00000075790	4
BCAT1	ENSG00000060982	5
BCL2L1	ENSG00000171552	4
BDP1	ENSG00000145734	4
BEGAIN	ENSG00000183092	4
BEND4	ENSG00000188848	5
BRCC3	ENSG00000185515	4
BRD3	ENSG00000169925	4
BRF2	ENSG00000104221	2
BRWD3	ENSG00000165288	4
BSDC1	ENSG00000160058	3
BSND	ENSG00000162399	3
BTBD3	ENSG00000132640	4
BZW1	ENSG00000082153	5
C11orf57	ENSG00000150776	4

C14orf178	ENSG00000197734	3
C14orf28	ENSG00000179476	5
C15orf39	ENSG00000167173	4
C15orf41	ENSG00000186073	4
C16orf52	ENSG00000185716	4
C18orf21	ENSG00000141428	4
C1GALT1	ENSG00000106392	4
C1orf112	ENSG00000000460	2
C1orf159	ENSG00000131591	4
C1orf200	ENSG00000179840	2
C1QTNF3AMACR	ENSG00000273294	3
C20orf194	ENSG00000088854	4
C3orf38	ENSG00000179021	4
C5orf51	ENSG00000205765	4
C8orf58	ENSG00000241852	4
C9orf40	ENSG00000135045	5
CALN1	ENSG00000183166	2
CAMK1	ENSG00000134072	4
CAPRIN2	ENSG00000110888	5
CASP3	ENSG00000164305	4
CASP5	ENSG00000137757	2
CBX5	ENSG00000094916	5
CCDC71L	ENSG00000253276	5
CCL7	ENSG00000108688	4
CCND2	ENSG00000118971	4
CCNF	ENSG00000162063	5
CCNJ	ENSG00000107443	5
CCR7	ENSG00000126353	4
CD200R1	ENSG00000163606	4
CD86	ENSG00000114013	4
CDC25A	ENSG00000164045	2
CDC34	ENSG00000099804	5
CDH8	ENSG00000150394	5

CDKN1A	ENSG00000124762	3
CDV3	ENSG00000091527	4
CEP85L	ENSG00000111860	4
CERCAM	ENSG00000167123	5
CHIA	ENSG00000134216	3
CHRNA7	ENSG00000175344	4
CHSY3	ENSG00000198108	4
CLCN5	ENSG00000171365	5
CLDN12	ENSG00000157224	5
CLDN16	ENSG00000113946	3
CLOCK	ENSG00000134852	4
CNOT2	ENSG00000111596	5
CNTRL	ENSG00000119397	5
COIL	ENSG00000121058	5
COL15A1	ENSG00000204291	4
COL1A1	ENSG00000108821	4
COL1A2	ENSG00000164692	5
COL27A1	ENSG00000196739	5
COL3A1	ENSG00000168542	5
COL4A1	ENSG00000187498	4
COL4A6	ENSG00000197565	4
COL5A2	ENSG00000204262	4
CPA4	ENSG00000128510	5
CPD	ENSG00000108582	4
CPEB1	ENSG00000214575	5
CPEB2	ENSG00000137449	4
CPEB3	ENSG00000107864	4
CPEB4	ENSG00000113742	4
CPM	ENSG00000135678	5
CRCT1	ENSG00000169509	5
CRTAP	ENSG00000170275	3
CSNK2A1	ENSG00000101266	4
CSTA	ENSG00000121552	4
CTD-2535L24.2	ENSG00000266076	5

CTDSPL2	ENSG00000137770	5
CTNND2	ENSG00000169862	4
CTPS2	ENSG00000047230	5
CTSA	ENSG00000064601	4
CYB561D1	ENSG00000174151	4
CYP19A1	ENSG00000137869	4
CYP2C8	ENSG00000138115	3
CYP2R1	ENSG00000186104	5
CYP4F2	ENSG00000186115	4
CYSLTR1	ENSG00000173198	2
DAB1	ENSG00000173406	4
DAGLA	ENSG00000134780	5
DCAF15	ENSG00000132017	4
DCAF6	ENSG00000143164	2
DCLRE1B	ENSG00000118655	2
DCLRE1C	ENSG00000152457	5
DCUN1D3	ENSG00000188215	4
DDI2	ENSG00000197312	5
DDX19B	ENSG00000157349	5
DDX26B	ENSG00000165359	5
DIAPH2	ENSG00000147202	5
DICER1	ENSG00000100697	5
DLC1	ENSG00000164741	4
DLGAP4	ENSG00000080845	5
DMD	ENSG00000198947	5
DMP1	ENSG00000152592	3
DNA2	ENSG00000138346	5
DNAJA2	ENSG00000069345	4
DNAJC10	ENSG00000077232	4
DOK3	ENSG00000146094	4
DPH3	ENSG00000154813	5
DSCR8	ENSG00000198054	4
DTX2	ENSG00000091073	4
DUSP1	ENSG00000120129	4

DUSP12	ENSG00000081721	3
DUSP22	ENSG00000112679	5
DVL3	ENSG00000161202	4
DYRK2	ENSG00000127334	4
DZIP1	ENSG00000134874	2
E2F2	ENSG00000007968	5
E2F5	ENSG00000133740	5
E2F6	ENSG00000169016	4
EDEM3	ENSG00000116406	4
EDN1	ENSG00000078401	5
EEF2K	ENSG00000103319	2
EFHD2	ENSG00000142634	4
EGLN2	ENSG00000269858	4
EIF4ENIF1	ENSG00000184708	5
EIF4G2	ENSG00000110321	5
EIF5B	ENSG00000158417	4
ELF4	ENSG00000102034	4
ELK4	ENSG00000158711	3
ENY2	ENSG00000120533	3
EOGT	ENSG00000163378	4
EPHA4	ENSG00000116106	4
EPHB1	ENSG00000154928	4
ERCC6	ENSG00000225830	5
ERO1L	ENSG00000197930	5
ERRFI1	ENSG00000116285	4
ESRP2	ENSG00000103067	4
ETV3	ENSG00000117036	4
EVI2B	ENSG00000185862	4
FAM103A1	ENSG00000169612	5
FAM104A	ENSG00000133193	4
FAM118A	ENSG00000100376	4
FAM135A	ENSG00000082269	4
FAM135B	ENSG00000147724	4
FAM178A	ENSG00000119906	5

FAM189A1	ENSG00000104059	4
FAM214A	ENSG00000047346	5
FAM214B	ENSG00000005238	4
FAM222B	ENSG00000173065	5
FAM71D	ENSG00000172717	2
FAS	ENSG00000026103	4
FAXC	ENSG00000146267	5
FBXL12	ENSG00000127452	5
FBXO15	ENSG00000141665	2
FGD6	ENSG00000180263	4
FGF11	ENSG00000161958	4
FGFR1OP	ENSG00000213066	4
FIGN	ENSG00000182263	5
FKTN	ENSG00000106692	4
FLNA	ENSG00000196924	4
FMO2	ENSG00000094963	4
FNDC3A	ENSG00000102531	5
FNIP1	ENSG00000217128	5
FOXP2	ENSG00000128573	5
FRAS1	ENSG00000138759	5
FRMD4B	ENSG00000114541	5
FRMD5	ENSG00000171877	4
FZD3	ENSG00000104290	4
FZD4	ENSG00000174804	5
GABRA6	ENSG00000145863	4
GALC	ENSG00000054983	4
GALE	ENSG00000117308	4
GALNT1	ENSG00000141429	5
GALNT16	ENSG00000100626	4
GAN	ENSG00000261609	5
GAREM	ENSG00000141441	4
GAS7	ENSG00000007237	5
GATM	ENSG00000171766	5
GCNT4	ENSG00000176928	5

GDAP2	ENSG00000196505	5
GDF6	ENSG00000156466	5
GDPD1	ENSG00000153982	4
GHR	ENSG00000112964	4
GIMAP6	ENSG00000133561	4
GIPC1	ENSG00000123159	4
GJC1	ENSG00000182963	4
GNG5	ENSG00000174021	5
GOLGA4	ENSG00000144674	4
GOLGA6L4	ENSG00000184206	4
GOLT1B	ENSG00000111711	5
GPAM	ENSG00000119927	3
GPATCH2	ENSG00000092978	4
GPATCH8	ENSG00000186566	3
GPC4	ENSG00000076716	5
GPCPD1	ENSG00000125772	4
GPR157	ENSG00000180758	5
GPR26	ENSG00000154478	5
GPR61	ENSG00000156097	4
GREB1L	ENSG00000141449	3
GRHL3	ENSG00000158055	4
GRID2IP	ENSG00000215045	3
GTF2I	ENSG00000077809	4
GXYLT1	ENSG00000151233	5
GYG2	ENSG00000056998	5
HABP4	ENSG00000130956	4
HAND1	ENSG00000113196	5
HDLBP	ENSG00000115677	4
HDX	ENSG00000165259	5
HECTD2	ENSG00000165338	4
HELLS	ENSG00000119969	4
HELZ	ENSG00000198265	4
HFE	ENSG00000010704	4
HIC2	ENSG00000169635	5
HIF1AN	ENSG00000166135	5

HIF3A	ENSG00000124440	5
HIP1	ENSG00000127946	5
HMGA1	ENSG00000137309	4
HOMER2	ENSG00000103942	4
HOOK1	ENSG00000134709	2
HOXA1	ENSG00000105991	5
HOXA9	ENSG00000078399	4
HOXB1	ENSG00000120094	5
HSPA14	ENSG00000187522	4
ICOS	ENSG00000163600	4
IDE	ENSG00000119912	3
IFI44L	ENSG00000137959	2
IFITM10	ENSG00000244242	5
IGDCC3	ENSG00000174498	5
IGDCC4	ENSG00000103742	5
IGF1	ENSG00000017427	4
IGF1R	ENSG00000140443	5
IGF2BP1	ENSG00000159217	5
IGF2BP2	ENSG00000073792	4
IGF2BP3	ENSG00000136231	5
IGLON5	ENSG00000142549	4
IGSF1	ENSG00000147255	4
IKBKE	ENSG00000143466	3
IL10	ENSG00000136634	5
IL13	ENSG00000169194	4
IL22RA1	ENSG00000142677	3
IL6R	ENSG00000160712	4
IMPDH1	ENSG00000106348	4
IMPG2	ENSG00000081148	5
ING3	ENSG00000071243	5
INO80D	ENSG00000114933	4
INSL4	ENSG00000120211	5
INSR	ENSG00000171105	4
INTS2	ENSG00000108506	4

INTS6	ENSG00000102786	5
IRGQ	ENSG00000167378	4
IRS2	ENSG00000185950	5
ITGA7	ENSG00000135424	4
ITGB6	ENSG00000115221	4
KATNAL1	ENSG00000102781	2
KATNBL1	ENSG00000134152	4
KCNE3	ENSG00000175538	2
KCNQ4	ENSG00000117013	4
KCNT1	ENSG00000107147	5
KCTD17	ENSG00000100379	5
KCTD2	ENSG00000180901	4
KCTD21	ENSG00000188997	5
KHNYN	ENSG00000100441	5
KIAA1161	ENSG00000164976	2
KIAA1958	ENSG00000165185	2
KLHDC8B	ENSG00000185909	5
KLHL31	ENSG00000124743	5
KLHL6	ENSG00000172578	3
KMT2D	ENSG00000167548	5
KMT2E	ENSG00000005483	4
KPNA5	ENSG00000196911	4
KRT5	ENSG00000186081	4
LA16c-431H6.6	ENSG00000261732	5
LBR	ENSG00000143815	4
LCOR	ENSG00000196233	4
LCORL	ENSG00000178177	5
LIMD2	ENSG00000136490	5
LIN28A	ENSG00000131914	5
LIN28B	ENSG00000187772	5
LINGO1	ENSG00000169783	4
LIPH	ENSG00000163898	4
LOR	ENSG00000203782	4
LOXL3	ENSG00000115318	3

LPGAT1	ENSG00000123684	5
LPHN3	ENSG00000150471	4
LRIG2	ENSG00000198799	5
LRP8	ENSG00000157193	5
LRRC17	ENSG00000128606	4
LRRC23	ENSG00000010626	5
LRTOMT	ENSG00000184154	4
LTN1	ENSG00000198862	4
LYVE1	ENSG00000133800	2
MACF1	ENSG00000127603	3
MAGEA6	ENSG00000197172	4
MAP3K1	ENSG00000095015	5
MAP4K2	ENSG00000168067	4
MAP4K3	ENSG00000011566	5
MAP4K4	ENSG00000071054	4
MAPK6	ENSG00000069956	5
MAPK8	ENSG00000107643	5
MARS2	ENSG00000247626	5
MASP1	ENSG00000127241	2
MBD2	ENSG00000134046	5
MCCC2	ENSG00000131844	2
MDM4	ENSG00000198625	5
MED28	ENSG00000118579	5
MED8	ENSG00000159479	4
MEF2C	ENSG00000081189	4
MEIS2	ENSG00000134138	3
METTL14	ENSG00000145388	4
MEX3A	ENSG00000254726	4
MFSD12	ENSG00000161091	4
MFSD4	ENSG00000174514	5
MGA	ENSG00000174197	4
MGAT4A	ENSG00000071073	5
MIB1	ENSG00000101752	2
MLLT10	ENSG00000078403	4

MRS2	ENSG00000124532	5
MSH6	ENSG00000116062	2
MTDH	ENSG00000147649	4
MXD1	ENSG00000059728	5
MYB	ENSG00000118513	4
MYCBP	ENSG00000214114	4
MYCN	ENSG00000134323	5
MYO18A	ENSG00000196535	3
NAA30	ENSG00000139977	5
NAP1L1	ENSG00000187109	5
NCR3LG1	ENSG00000188211	5
NDST2	ENSG00000166507	4
NDST3	ENSG00000164100	5
NDUFA4	ENSG00000189043	4
NEDD4	ENSG00000069869	4
NEFM	ENSG00000104722	4
NEK3	ENSG00000136098	4
NFAT5	ENSG00000102908	5
NGF	ENSG00000134259	5
NHLRC3	ENSG00000188811	5
NID2	ENSG00000087303	4
NIPA1	ENSG00000170113	5
NIPAL4	ENSG00000172548	4
NME6	ENSG00000172113	5
NME9	ENSG00000181322	2
NOL4L	ENSG00000197183	5
NPIPA7	ENSG00000233024	4
NR6A1	ENSG00000148200	5
NRAS	ENSG00000213281	5
NT5C3L	ENSG00000141698	4
NTRK3	ENSG00000140538	4
NYNRIN	ENSG00000205978	5
OLFM4	ENSG00000102837	4
ONECUT2	ENSG00000119547	5

ONECUT3	ENSG00000205922	4
OSMR	ENSG00000145623	5
OSTF1	ENSG00000134996	4
OTOF	ENSG00000115155	4
P2RY13	ENSG00000181631	4
P4HA2	ENSG00000072682	3
PAG1	ENSG00000076641	4
PAK6	ENSG00000259288	4
PALD1	ENSG00000107719	4
PAPOLA	ENSG00000090060	2
PAPPA	ENSG00000182752	5
PARD6B	ENSG00000124171	5
PARP8	ENSG00000151883	5
PARS2	ENSG00000162396	4
PAWR	ENSG00000177425	4
PBX1	ENSG00000185630	5
PBX3	ENSG00000167081	5
PCBP4	ENSG00000090097	4
PCDH19	ENSG00000165194	5
PCGF3	ENSG00000185619	4
PCTP	ENSG00000141179	4
PCYOX1L	ENSG00000145882	5
PDE12	ENSG00000174840	5
PDPR	ENSG00000090857	4
PDZD9	ENSG00000155714	4
PGAP2	ENSG00000148985	4
PGRMC1	ENSG00000101856	5
PHACTR2	ENSG00000112419	4
PHLDB2	ENSG00000144824	4
PIK3IP1	ENSG00000100100	4
PLA2G3	ENSG00000100078	4
PLCB4	ENSG00000101333	2
PLEKHA8	ENSG00000106086	5
PLEKHG6	ENSG00000008323	5

PLEKHG7	ENSG00000187510	4
PLEKHO1	ENSG00000023902	4
PLS3	ENSG00000102024	4
PLXND1	ENSG00000004399	3
POGZ	ENSG00000143442	3
POLE	ENSG00000177084	3
POLR2D	ENSG00000144231	3
PORCN	ENSG00000102312	5
POTEE	ENSG00000188219	5
POTEG	ENSG00000222036	5
POTEM	ENSG00000187537	5
POU2F1	ENSG00000143190	5
POU2F2	ENSG00000028277	4
PPAPDC1B	ENSG00000147535	4
PPAPDC2	ENSG00000205808	4
PPARGC1A	ENSG00000109819	4
PPARGC1B	ENSG00000155846	5
PPP1R15B	ENSG00000158615	5
PPP4C	ENSG00000149923	5
PREB	ENSG00000138073	4
PRKAR2A	ENSG00000114302	5
PRLR	ENSG00000113494	3
PRPF38B	ENSG00000134186	4
PRR22	ENSG00000212123	3
PRR23A	ENSG00000206260	5
PRTG	ENSG00000166450	5
PTAFR	ENSG00000169403	5
PTAR1	ENSG00000188647	4
PTCH1	ENSG00000185920	5
PTCHD4	ENSG00000244694	3
PTPRD	ENSG00000153707	5
PTPRK	ENSG00000152894	5
PXDN	ENSG00000130508	4
PXT1	ENSG00000179165	5
RAB11FIP2	ENSG00000107560	4

RAB11FIP4	ENSG00000131242	2
RAB3GAP2	ENSG00000118873	4
RANBP2	ENSG00000153201	4
RASGRP1	ENSG00000172575	5
RBFOX1	ENSG00000078328	4
RBFOX2	ENSG00000100320	4
RBMS1	ENSG00000153250	5
RDX	ENSG00000137710	4
REV3L	ENSG00000009413	5
RFWD3	ENSG00000168411	2
RFX6	ENSG00000185002	5
RGS16	ENSG00000143333	5
RICTOR	ENSG00000164327	5
RIF1	ENSG00000080345	3
RIMKLA	ENSG00000177181	2
RIOK3	ENSG00000101782	4
RMDN2	ENSG00000115841	4
RMI2	ENSG00000175643	2
RNF165	ENSG00000141622	2
RNF170	ENSG00000120925	4
RNF217	ENSG00000146373	4
RNF44	ENSG00000146083	4
RNFT1	ENSG00000189050	5
RP11-108O10.8	ENSG00000258529	5
RP11-152F13.5	ENSG00000196648	4
RP11-574K11.31	ENSG00000272916	3
RPS6KB2	ENSG00000175634	5
RRM2	ENSG00000171848	4
RSPO2	ENSG00000147655	5
SCD	ENSG00000099194	5
SCN8A	ENSG00000196876	4
SCYL3	ENSG00000000457	4
SDR42E1	ENSG00000184860	4
SEC14L1	ENSG00000129657	4

SELT	ENSG00000198843	4
SEMA4C	ENSG00000168758	4
SENP2	ENSG00000163904	5
SENP5	ENSG00000119231	4
SESN3	ENSG00000149212	2
SESTD1	ENSG00000187231	4
SFMBT1	ENSG00000163935	4
SFSWAP	ENSG00000061936	5
SH3TC1	ENSG00000125089	4
SIGLEC14	ENSG00000254415	4
SKIDA1	ENSG00000180592	4
SKIL	ENSG00000136603	5
SLC16A10	ENSG00000112394	4
SLC16A9	ENSG00000165449	4
SLC20A1	ENSG00000144136	5
SLC22A23	ENSG00000137266	4
SLC25A18	ENSG00000182902	4
SLC25A24	ENSG00000085491	2
SLC25A27	ENSG00000153291	5
SLC2A12	ENSG00000146411	4
SLC30A1	ENSG00000170385	5
SLC30A4	ENSG00000104154	4
SLC30A6	ENSG00000152683	4
SLC35B1	ENSG00000121073	3
SLC35D2	ENSG00000130958	5
SLC38A2	ENSG00000134294	5
SLC38A9	ENSG00000177058	4
SLC5A11	ENSG00000158865	4
SLC5A6	ENSG00000138074	4
SLC5A9	ENSG00000117834	4
SLC6A5	ENSG00000165970	5
SLCO5A1	ENSG00000137571	4
SMAP1	ENSG00000112305	4
SMARCAD1	ENSG00000163104	5

SMARCC1	ENSG00000173473	5
SMC1A	ENSG00000072501	5
SMCHD1	ENSG00000101596	4
SMIM3	ENSG00000256235	4
SNAP23	ENSG00000092531	4
SNX16	ENSG00000104497	4
SNX25	ENSG00000109762	5
SNX27	ENSG00000143376	3
SOCS4	ENSG00000180008	4
SOX6	ENSG00000110693	4
SPAG16	ENSG00000144451	2
SPEG	ENSG00000072195	3
SRD5A3	ENSG00000128039	4
SREK1IP1	ENSG00000153006	4
SRGAP1	ENSG00000196935	2
SRGAP3	ENSG00000196220	2
SRL	ENSG00000185739	4
ST8SIA1	ENSG00000111728	5
STARD13	ENSG00000133121	4
STARD3	ENSG00000131748	2
STARD3NL	ENSG00000010270	4
STARD9	ENSG00000159433	3
STK24	ENSG00000102572	4
STRBP	ENSG00000165209	4
STX16-NPEPL1	ENSG00000254995	3
STX3	ENSG00000166900	5
STYX	ENSG00000198252	2
SVIL	ENSG00000197321	4
SWT1	ENSG00000116668	2
SYNC	ENSG00000162520	5
SYNCRIP	ENSG00000135316	5
SYT1	ENSG00000067715	4
SYT11	ENSG00000132718	4
SYT2	ENSG00000143858	4

SYT7	ENSG00000011347	4
TAF5	ENSG00000148835	4
TARBP2	ENSG00000139546	5
TBKBP1	ENSG00000198933	5
TECPR2	ENSG00000196663	4
TET2	ENSG00000168769	3
TET3	ENSG00000187605	4
TFAP2A	ENSG00000137203	4
TFEC	ENSG00000105967	2
TFR2	ENSG00000106327	4
TGFBR1	ENSG00000106799	5
THEM4	ENSG00000159445	2
THOC2	ENSG00000125676	4
THRSP	ENSG00000151365	4
TMC7	ENSG00000170537	2
TMED4	ENSG00000158604	4
TMEM135	ENSG00000166575	5
TMEM143	ENSG00000161558	4
TMEM167A	ENSG00000174695	4
TMEM194A	ENSG00000166881	4
TMEM211	ENSG00000206069	5
TMEM65	ENSG00000164983	4
TMOD2	ENSG00000128872	4
TMPPE	ENSG00000188167	5
TNFRSF1B	ENSG00000028137	4
TNFSF10	ENSG00000121858	2
TNFSF9	ENSG00000125657	5
TP53	ENSG00000141510	2
TPM2	ENSG00000198467	4
TPP1	ENSG00000166340	5
TRABD	ENSG00000170638	4
TRANK1	ENSG00000168016	5
TRAPPC1	ENSG00000170043	3
TRHDE	ENSG00000072657	4

TRIM67	ENSG00000119283	4
TRIM71	ENSG00000206557	5
TRIP12	ENSG00000153827	4
TRMT10A	ENSG00000145331	4
TRMT13	ENSG00000122435	4
TSC1	ENSG00000165699	4
TSPEAR	ENSG00000175894	4
TTC14	ENSG00000163728	3
TTC26	ENSG00000105948	5
TTC31	ENSG00000115282	5
TTL	ENSG00000114999	5
TTLL4	ENSG00000135912	5
TXLNG	ENSG00000086712	5
UFM1	ENSG00000120686	4
UGCG	ENSG00000148154	2
UGT3A1	ENSG00000145626	3
UHRF2	ENSG00000147854	5
ULK3	ENSG00000140474	5
USP12	ENSG00000152484	4
USP21	ENSG00000143258	4
USP24	ENSG00000162402	5
USP38	ENSG00000170185	5
USP44	ENSG00000136014	5
USP47	ENSG00000170242	4
UST	ENSG00000111962	3
UTRN	ENSG00000152818	5
VASH2	ENSG00000143494	3
VEZT	ENSG00000028203	3
VPS33A	ENSG00000139719	4
VSTM5	ENSG00000214376	5
WASL	ENSG00000106299	4
WBP1L	ENSG00000166272	2
WDFY4	ENSG00000128815	5
WDR11	ENSG00000120008	4

WNT9A	ENSG00000143816	5
XK	ENSG00000047597	5
XKR4	ENSG00000206579	5
XKR8	ENSG00000158156	5
XPOT	ENSG00000184575	4
XRN1	ENSG00000114127	4
XYLT2	ENSG00000015532	4
YOD1	ENSG00000180667	5
YY1	ENSG00000100811	3
ZBTB10	ENSG00000205189	3
ZBTB26	ENSG00000171448	5
ZBTB37	ENSG00000185278	2
ZC3H3	ENSG00000014164	4
ZCCHC9	ENSG00000131732	5
ZFHX4	ENSG00000091656	4
ZFYVE26	ENSG00000072121	5
ZNF10	ENSG00000256223	4
ZNF200	ENSG00000010539	4
ZNF202	ENSG00000166261	4
ZNF275	ENSG00000063587	4
ZNF280B	ENSG00000198477	5
ZNF341	ENSG00000131061	5
ZNF343	ENSG00000088876	3
ZNF347	ENSG00000197937	4
ZNF354B	ENSG00000178338	3
ZNF429	ENSG00000197013	4
ZNF432	ENSG00000256087	4
ZNF451	ENSG00000112200	3
ZNF462	ENSG00000148143	4
ZNF512B	ENSG00000196700	5
ZNF543	ENSG00000178229	3
ZNF577	ENSG00000161551	4
ZNF583	ENSG00000198440	4
ZNF620	ENSG00000177842	4
ZNF644	ENSG00000122482	5

ZNF689	ENSG00000156853	5
ZNF710	ENSG00000140548	5
ZNF738	ENSG00000172687	5
ZNF774	ENSG00000196391	4
ZNF784	ENSG00000179922	4
ZNF843	ENSG00000176723	4
ZNF880	ENSG00000221923	4
ZPLD1	ENSG00000170044	4
ZSWIM5	ENSG00000162415	4
